# Supplementary material for: T7 RNA polymerase-based gene expression from a transcriptionally silent rDNA spacer in the endosymbiont-harboring trypanosomatid Angomonas deanei
Source: PLoS One. 2025 May 30;20(5):e0322611. doi: 10.1371/journal.pone.0322611 (PMC12124550; doi:10.1371/journal.pone.0322611)
Supplement: S1 Appendix — Regions flanking the insertion site that were used for homologous recombination are highlighted in yellow (3’ flank) and red (5’ flank). rDNA sequences in A. deanei were identified using the rDNA sequence from T. brucei as query (NCBI locus tags TB927_01.rRNA.1 and onwards). The start of the rDNA arrays is marked with “Start of 18S rDNA” at the end of the alignment. For the multi-sequence alignment Clustal Omega was used (www.ebi.ac.uk/jdispatcher/msa/clustalo). (PDF) [file pone.0322611.s001.pdf]

Fig. 2

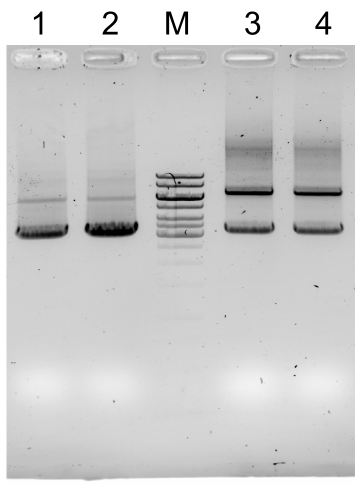

1: *A. deanei* WT
2: *A. deanei* WT
M: 1 kB GeneRuler (ThermoFisher Scientific)
3: Adea319 (C1)
4: Adea319 (C2)

Detection method: TAE agarose gel with PCR samples, detection with "SYBR Safe" filter for nucleic acids (ChemiDoc MP, Biorad)

Derived figure in manuscript: 2B

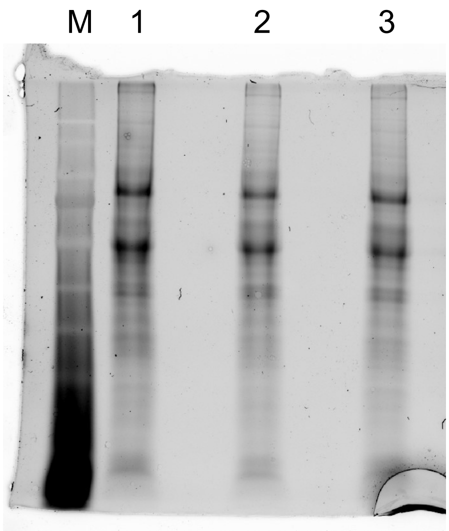

M: PAGE Ruler prestained (ThermoFisher Scientific)
1: *A. deanei* WT
2: Adea319
2: Adea400

Detection method: SDS-PAGE containing TCE with protein samples, detection with "Stainfree gel" settings (ChemiDoc MP, Biorad)

Derived figure in manuscript: 2C (left)

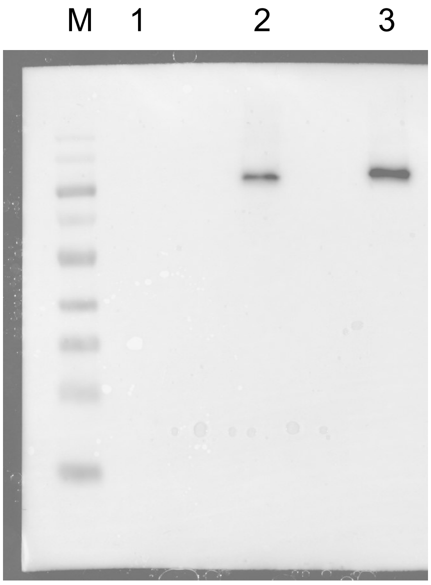

M: PAGE Ruler prestained (ThermoFisher Scientific)
1: *A. deanei* WT
2: Adea319
2: Adea400

Detection method: Western Blot targeting T7 RNA polymerase, detection with "Chemiluminescence" settings (ChemiDoc MP, Biorad)

Derived figure in manuscript: 2C (right)

Fig. 4

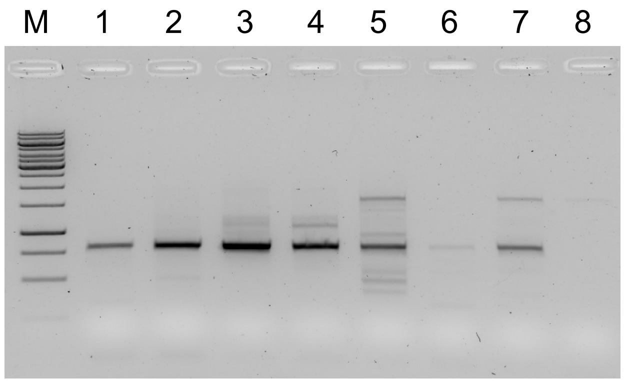

M: 1 kB GeneRuler (ThermoFisher Scientific)
1-7: Adea400 (C1-C7)
8: *A. deanei* WT

Detection method: TAE agarose gel with PCR samples, detection with "SYBR Safe" filter for nucleic acids (ChemiDoc MP, Biorad)

Derived figure in manuscript: 4B

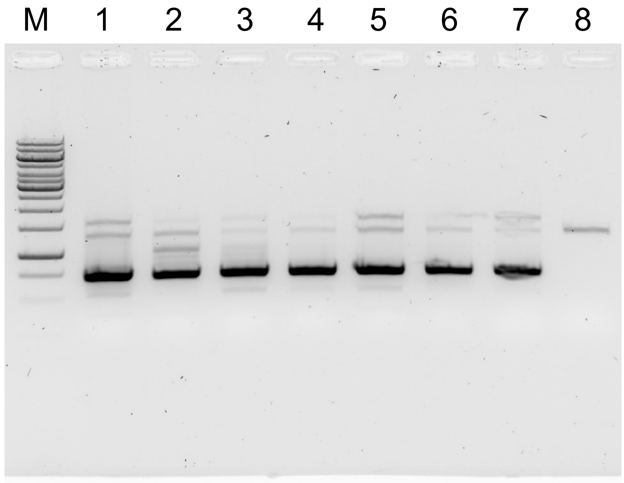

M: 1 kB GeneRuler (ThermoFisher Scientific)
1-7: Adea400 (C1-C7)
8: *A. deanei* WT

Detection method: TAE agarose gel with PCR samples, detection with "SYBR Safe" filter for nucleic acids (ChemiDoc MP, Biorad)

Derived figure in manuscript: 4C

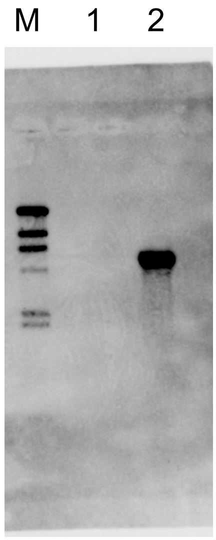

M: DNA Molecular Weight Marker II (Roche)
1: *A. deanei* WT
2: Adea400

Detection method: Southern Blot targeting *blast*^{R}, detection with "Chemiluminescence" settings (ChemiDoc MP, Biorad)

Derived figure in manuscript: 4D

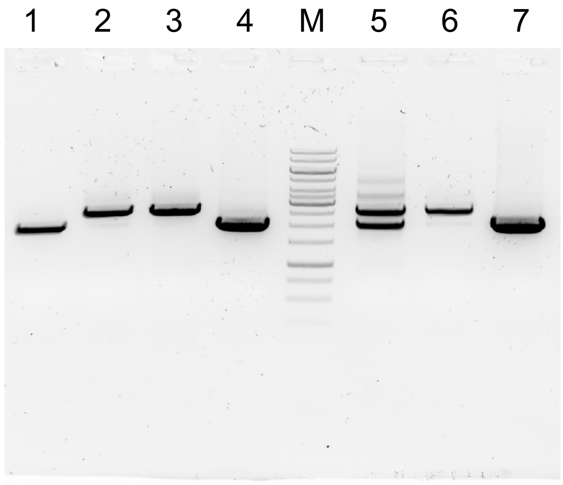

M: 1 kB GeneRuler (ThermoFisher Scientific)
1-6: Adea442 (C1-C6) -> T7RNAP KO-strain
8: Adea319

Detection method: TAE agarose gel with PCR samples, detection with "SYBR Safe" filter for nucleic acids (ChemiDoc MP, Biorad)

Derived figure in manuscript: 4G

Fig. 5

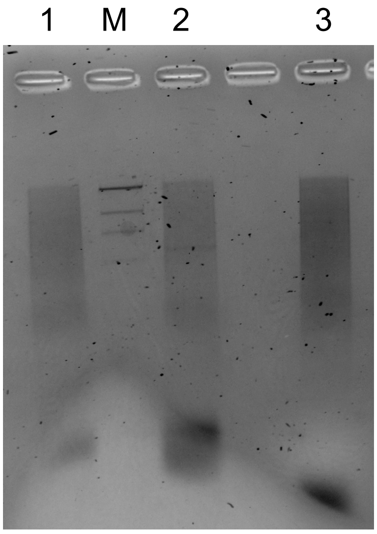

1: *A. deanei* WT
M: DNA Molecular Weight Marker II (Roche)
2: Adea400
3: Adea456

Detection method: Southern Blot targeting *blast*^{R}, detection with "Chemiluminescence" settings (ChemiDoc MP, Biorad)

Derived figure in manuscript: 5B (top)

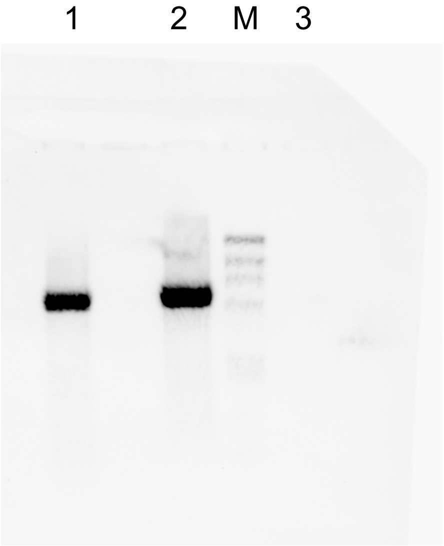

1: Adea456
2: Adea400
M: DNA Molecular Weight Marker II (Roche)
3: *A. deanei* WT

Detection method: Southern Blot targeting *blast*^{R}, detection with "Chemiluminescence" settings (ChemiDoc MP, Biorad)

Derived figure in manuscript: 5B (bottom)

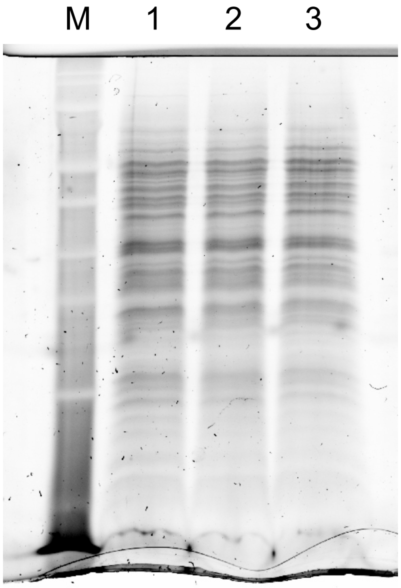

M: PAGE Ruler prestained (ThermoFisher Scientific)
1: *A. deanei* WT
2: Adea126
3: Adea456

Detection method: SDS-PAGE containing TCE with protein samples, detection with "Stainfree gel" settings (ChemiDoc MP, Biorad)

Derived figure in manuscript: 5F (top)

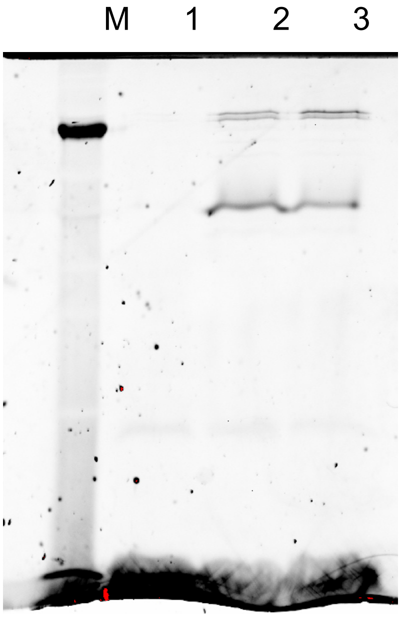

M: PAGE Ruler prestained (ThermoFisher Scientific)
1: *A. deanei* WT
2: Adea126
3: Adea456

Detection method: In gel fluorescence assay with proteins detecting mScarlet, detection with "Cy3" filter (ChemiDoc MP, Biorad)

Derived figure in manuscript: 5F (bottom)

Supp.

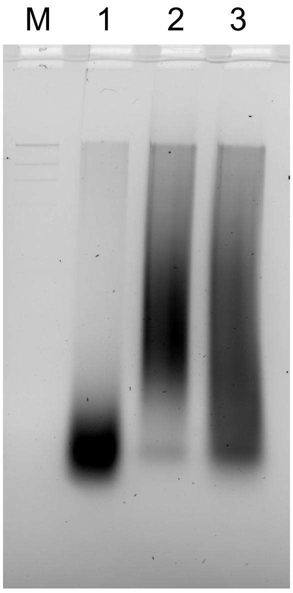

M: DNA Molecular Weight Marker II (Roche)
1: *A. deanei* WT
2: Adea400
3: Adea456

Detection method: Southern Blot targeting *blast*^{R}, detection with "Chemiluminescence" settings (ChemiDoc MP, Biorad)

Derived figure in manuscript: S1 Figure (top)

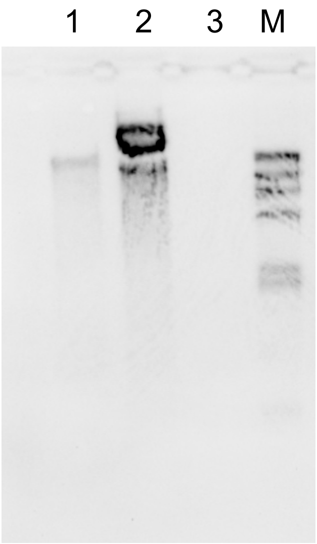

M: DNA Molecular Weight Marker II (Roche)
1: Adea456
2: Adea400
3: *A. deanei* WT

Detection method: Southern Blot targeting *blast*^{R}, detection with "Chemiluminescence" settings (ChemiDoc MP, Biorad)

Derived figure in manuscript: S1 Figure (bottom)
