# Supplementary figures and images for: T7 RNA polymerase-based gene expression from a transcriptionally silent rDNA spacer in the endosymbiont-harboring trypanosomatid Angomonas deanei
Source: PLoS One. 2025 May 30;20(5):e0322611. doi: 10.1371/journal.pone.0322611 (PMC12124550; doi:10.1371/journal.pone.0322611)

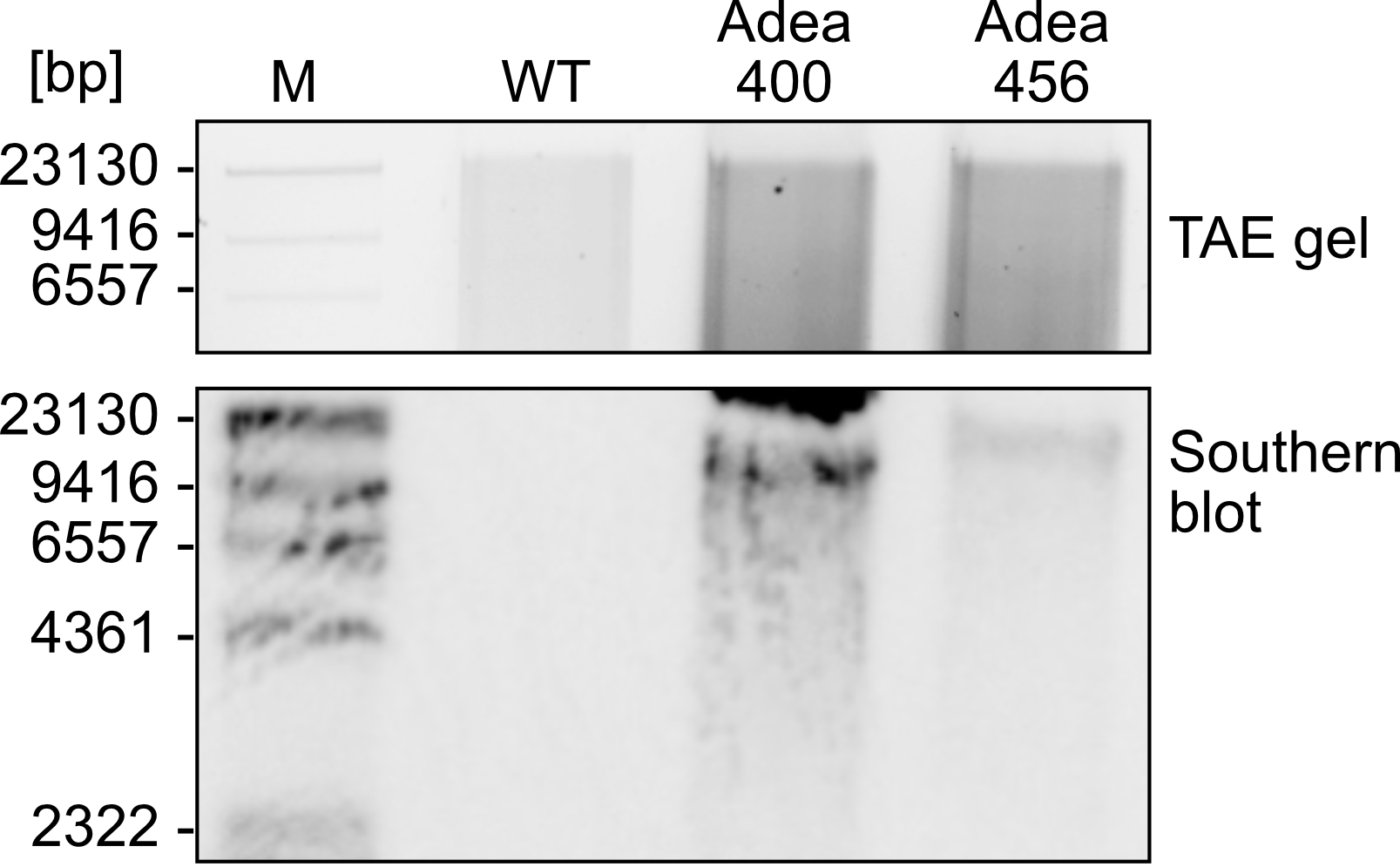

Supplement: S1 Fig — The WT served as control. The blastR-targeting probe yielded single bands of the expected sizes for Adea400 (16252 bp) and Adea456 (18519 bp) but not for the WT (see S1 Table for expected sizes). (TIF) [file pone.0322611.s002.tif]

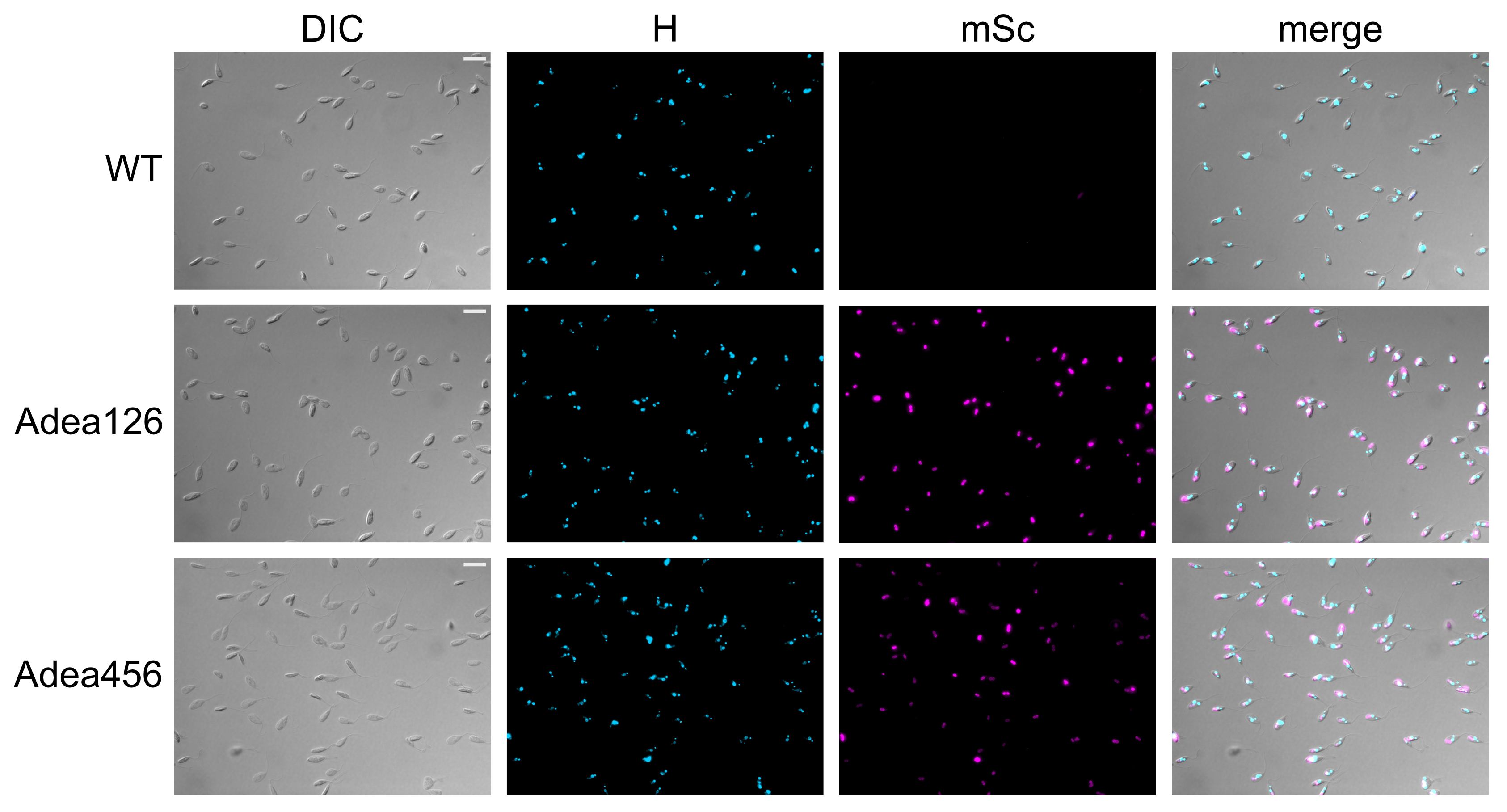

Supplement: S2 Fig — DIC, differential interference contrast; H, blue channel (visualizing Hoechst 33342-stained DNA); mSc, red channel (visualizing mScarlet). Scale bar represents 10 µm. (TIF) [file pone.0322611.s003.tif]
