# Supplementary material for: T7 RNA polymerase-based gene expression from a transcriptionally silent rDNA spacer in the endosymbiont-harboring trypanosomatid Angomonas deanei
Source: PLoS One. 2025 May 30;20(5):e0322611. doi: 10.1371/journal.pone.0322611 (PMC12124550; doi:10.1371/journal.pone.0322611)
Supplement: S1 Table — For the identification of the rDNA spacer, in which transgenes had been inserted, genomic DNA of the relevant strains was digested using a combination of BclI and SpeI or BamHI alone. (PDF) [file pone.0322611.s004.pdf]

S1 Table. Expected Southern blot fragments for the verification of Adea400 and Adea456. For the identification of the rDNA spacer, in which transgenes had been inserted, genomic DNA of the relevant strains was digested using a combination of BclI and SpeI or BamHI alone.

	**Length of expected band [bp]**			
**Strain**	**Adea400**		**Adea456**	
**Chromosome**	**SpeI+BclI**	**BamHI**	**SpeI+BclI**	**BamHI**
13	4,746	16,252	4,345	18,519
3	8,142	4,535	4,332	6,802
6	>30,000	5,520	4,353	7,787
17	7,926	5,515	4,347	7,782
25	4,943	7,453	4,355	9,720
27	10,999	5,525	4,356	7,792
29	14,036	8,131	4,356	10,398
